# Supplementary material for: Content-rich biological network constructed by mining PubMed abstracts
Source: BMC Bioinformatics. 2004 Oct 8;5:147. doi: 10.1186/1471-2105-5-147 (PMC528731; doi:10.1186/1471-2105-5-147)
Supplement: Additional File 2 — The original results of the above study (non-essential files are deleted to keep the file size under the limit set by BMC bioinformatics). [file 1471-2105-5-147-S2.bz2 › chilibotAdditionalFile2/dip40/14ID9153399E59/html/ITGB2_ICAM2.html]

 


 **ITGB2** and **ICAM2** 
  
Found 158 abstracts in PubMed, retrieved 40.  
 

 What does Google say? 
 PDF only 
| .edu only 

---

**Interactive relationship** (e.g. stimulation, inhibition, etc)

**Neutral relationship**- Previous findings have shown that a peptide derived from the first Ig domain of ICAM 2  [ **ICAM2** ]  P1 binds to LFA 1  [ **ITGB2** ]  CD11a CD18  [ **ITGB2** ]  and Mac 1 CD11b CD18  [ **ITGB2** ]  and activates leukocyte aggregation.  Ref: 10352278 J Immunol, 1999
- The CD11b CD18  [ **ITGB2** ]  macrophage receptor can also bind to ICAM 1 and ICAM 2  [ **ICAM2** ] .  Ref: 10360689 J Bone Joint Surg Am, 1999
- Consistent with this observation, immobilized, purified ICAM 2  [ **ICAM2** ] , a specific counter receptor for LFA 1  [ **ITGB2** ] , did not support the adhesion dependent phase of superoxide production.  Ref: 11073107 J Leukoc Biol, 2000
- The results indicate that 1 LFA 1  [ **ITGB2** ]  mediates PMN TEM primarily by interaction with ICAM 1 and ICAM 2  [ **ICAM2** ] .  Ref: 9886254 J Leukoc Biol, 1999
- We show that this process is dependent on an initial interaction between alphaLbeta2 integrin CD11a CD18  [ **ITGB2** ]  on infected monocytic cells and intercellular adhesion molecule 2  [ **ICAM2** ]  ICAM 2  [ **ICAM2** ] .  Ref: 11739669 J Virol, 2002
- The LFA 1  [ **ITGB2** ]  adhesion molecule is involved in cell adhesion events of leukocytes through binding to ICAM 1, ICAM 2  [ **ICAM2** ]  and ICAM 3.  Ref: 10427988 Eur J Immunol, 1999
- The beta 2 integrin LFA 1  [ **ITGB2** ]  CD11a CD18  [ **ITGB2** ]  is a leukocyte specific adhesion molecule that mediates leukocyte extravasation, antigen presentation, and T cell mediated cytolysis through its interaction with its counter receptors, ICAM 1, ICAM 2  [ **ICAM2** ] , and ICAM 3.  Ref: 12799043 J Immunol Methods, 2003
- An antibody against intercellular adhesion molecule ICAM 2  [ **ICAM2** ]  gave an inhibition similar to that by anti CD18  [ **ITGB2** ]  mAb, suggesting ICAM 2  [ **ICAM2** ]  was the major counter receptor involved for the beta2 integrin component.  Ref: 10229100 Eur J Immunol, 1999
- Interestingly, we observed that LFA 1  [ **ITGB2** ]  ICAM 1 co stimulation of T cells resulted in high production of the Th2 cytokine IL 10 compared to ICAM 2  [ **ICAM2** ]  or ICAM 3 co stimulation.  Ref: 10427988 Eur J Immunol, 1999

**Non-interactive relationship** (e.g. studied together, co-existance, homology, etc.)

- ICAM 2  [ **ICAM2** ]  engagement by CD19 cells of its natural receptor, LFA 1  [ **ITGB2** ] , on CD4 naive cells specifically induced AKT activity in the absence of an MHC peptide interaction.  Ref: 11825565 Immunity, 2002
- Agglutination induced by A80 was inhibited by stromal cell derived factor 1, T22, and phorbol 12 myristate 13 acetate but was not significantly altered by pretreatment of cells with pertussis toxin, wortmannin, or MAbs to LFA 1  [ **ITGB2** ] , ICAM 1, ICAM 2  [ **ICAM2** ] , and ICAM 3.  Ref: 11689635 J Virol, 2001
- Serial colonic sections 10 UC, 10 CD, and 10 controls were stained immunohistochemically for ICAM 1, ICAM 2  [ **ICAM2** ] , ICAM 3, CD11a, CD11b, CD18  [ **ITGB2** ] , and PECAM 1.  Ref: 10935652 Am J Surg Pathol, 2000
- Role of ICAM 1 and ICAM 2  [ **ICAM2** ]  and alternate CD11 CD18  [ **ITGB2** ]  ligands in neutrophil transendothelial migration.  Ref: 9886254 J Leukoc Biol, 1999
- ICAM 2  [ **ICAM2** ] , a constitutively expressed endothelial ligand for beta2 integrins LFA 1  [ **ITGB2** ]  and Mac 1, is involved in leukocyte adhesion to resting endothelium and in transmigration in vitro, however its role in inflammation is unclear.  Ref: 10223354 Cell Adhes Commun, 1998
- L selectin expression decreased at 6 and 24 h, but ICAM 2  [ **ICAM2** ] , ICAM 3, LFA 1  [ **ITGB2** ] , and CD29 expression did not show any significant change after calcium ionophore stimulation.  Ref: 10688433 Allergy, 1999
- They expressed CD4, CD11a, CD11b, CD11c, CD18  [ **ITGB2** ] , CD32, CD33, CD45R0, CD48, CD50, CD54, CD58, CD64, CD86, CD102  [ **ICAM2** ] , CD116, CD123, and HLA DR.  Ref: 11698124 Exp Hematol, 2001
- We evaluated the relative contribution of ICAM 1 and ICAM 2  [ **ICAM2** ] , known ligands on endothelium for LFA 1  [ **ITGB2** ]  and Mac 1, in spontaneous neutrophil PMN transendothelial migration TEM across IL 1 activated HUVEC monolayers or TEM induced by C5a or IL 8 across unstimulated HUVEC grown on polycarbonate filters.  Ref: 9886254 J Leukoc Biol, 1999
- Although the integrin LFA 1  [ **ITGB2** ]  is a counter receptor for both ICAM 2  [ **ICAM2** ]  and ICAM 3, on DC, DC SIGN is the high affinity adhesion receptor for ICAM 2  [ **ICAM2** ]  3.  Ref: 12234361 Immunol Rev, 2002
- Adhesion molecules identified were intercellular adhesion molecule 1 ICAM 1, ICAM 2  [ **ICAM2** ] , ICAM 3, vascular cell adhesion molecule 1 VCAM 1, platelet endothelial cell adhesion molecule 1 PECAM 1, E selectin, P selectin, L selectin, lymphocyte function associated antigen 1 LFA 1  [ **ITGB2** ] , very late activation antigen 4 VLA 4, Mac 1 CD18  [ **ITGB2** ]  CD11b, and gp 15 5 CD18  [ **ITGB2** ]  CD11c.  Ref: 10643715 Arthritis Rheum, 2000
- For natural killer cell mediated cytolysis NK lysis and lymphokine activated killer cell mediated cytolysis LAK lysis, the co stimulatory signals of CD18  [ **ITGB2** ]  CD54 CD102  [ **ICAM2** ]  and CD2 CD58 pathways are essential.  Ref: 11061342 Oncol Res, 2000
- Pretreatment of human PMN with anti CD18  [ **ITGB2** ]  mAb or pretreatment of HUVEC with a combination of three mAbs against ICAM 1, ICAM 2  [ **ICAM2** ] , and E selectin inhibited by 85% the MC dependent PMN transmigration.  Ref: 12370389 J Immunol, 2002
- CBDC expressed CD4, CD11a, CD18  [ **ITGB2** ] , CD45RA, CD50, CD54, and CD123, but showed no expression of CD1a, CD11c, CD33, CD40, CD45R0, CD80, CD83, and CD86 and only limited expression of CD58, CD102  [ **ICAM2** ] , and CD116.  Ref: 10090940 Blood, 1999
- All the cytokines tested interleukin 1beta IL 1beta, IL 2, IL 4, IL 7, IL 10, interferon gamma IFN gamma as well as CAMs ICAM 1, ICAM 2  [ **ICAM2** ] , VCAM 1, LFA 1  [ **ITGB2** ]  and CD44 were expressed at substantial levels in both spleen and LNs.  Ref: 12193230 Scand J Immunol, 2002
- In conclusion, CD11b, CD18  [ **ITGB2** ] , and ICAM 2  [ **ICAM2** ]  seem to be important for PMN transepithelial migration in UC.  Ref: 10935652 Am J Surg Pathol, 2000
- Blocking monoclonal antibodies were used against the platelet and neutrophil surface receptors glycoprotein complex IIb IIla, P selectin, ICAM 2  [ **ICAM2** ] , CD11a, CD11b, and CD18  [ **ITGB2** ] .  Ref: 12069189 Shock, 2002
- RESULTS Peripheral blood eosinophils constitutively expressed low levels of ICAM 1 and ICAM 2  [ **ICAM2** ]  < ; 10 FMI, moderate levels 10 50 FMI of CD29 and L selectin, and high levels >50 FMI of ICAM 3, LFA 1  [ **ITGB2** ] , and Mac 1.  Ref: 10688433 Allergy, 1999
- Linear and cyclic peptides derived from the sequences of LFA 1  [ **ITGB2** ] , ICAM 1, ICAM 2  [ **ICAM2** ] , VCAM 1, and FN C1 have been shown to have inhibitory effects in vitro and in vivo.  Ref: 11857637 Med Res Rev, 2002
- LFA 1  [ **ITGB2** ]  and its ligands, ICAM 1, ICAM 2  [ **ICAM2** ] , and ICAM 3, can be expressed on the cells infected by HIV, as well as on the HIV virions themselves.  Ref: 11134324 J Virol, 2001
- CD18  [ **ITGB2** ]  CD54 CD102  [ **ICAM2** ] , CD2 CD58 pathway independent killing of lymphokine activated killer LAK cells against glioblastoma cell lines T98G and U373MG.  Ref: 11061342 Oncol Res, 2000
- In contrast, ICAM 2  [ **ICAM2** ]  and ICAM 3 induced a much stronger secretion of the Th1 cytokine TNF alpha compared to LFA 1  [ **ITGB2** ]  ICAM 1 induced co stimulation, despite the lower proliferation rate.  Ref: 10427988 Eur J Immunol, 1999
- IL 1beta is a co stimulator in activating Th cells, which manifests its activity together with LFA 1  [ **ITGB2** ] , ICAM 1 and ICAM 2  [ **ICAM2** ] .  Ref: 12400871 Int Immunopharmacol, 2002
- ICAM 1 antibody prevented the extravasation, while antibodies to other adhesion molecules showed little CD18  [ **ITGB2** ] , ICAM 2  [ **ICAM2** ]  or no inhibition CD11a and ICAM 3.  Ref: 11442494 Br J Haematol, 2001
- Based on this crystal structure, we mutagenized ICAM 2  [ **ICAM2** ]  to localize the binding site for the integrin lymphocyte function associated antigen 1 LFA 1  [ **ITGB2** ] .  Ref: 10077629 Proc Natl Acad Sci U S A, 1999
- Here we show that cross linking of part of the LFA 1  [ **ITGB2** ]  molecules with Abs causes activation of free LFA 1  [ **ITGB2** ]  molecules not occupied by the Ab on the same cell, which then bind to ICAM 2  [ **ICAM2** ]  on other cells.  Ref: 10510363 J Immunol, 1999
- These findings suggested that this type of the LAK lysis might not depend upon the CD18  [ **ITGB2** ]  CD54 CD102  [ **ICAM2** ]  pathway or CD2 CD58 pathway.  Ref: 11061342 Oncol Res, 2000
- To account for the different effects of CD11a and CD54 blockade in vivo, an additional CD11a CD18  [ **ITGB2** ]  ligand, CD102  [ **ICAM2** ]  ICAM 2  [ **ICAM2** ] , was demonstrated on tumor associated macrophages but not on tumor cells.  Ref: 10087180 Cell Immunol, 1999
- RESULTS Specific cation dependent binding of lymphocytes to bronchial endothelium was observed which was significantly inhibited by antibodies against P selectin, PSGL 1, L selectin, LFA 1  [ **ITGB2** ] , ICAM 1 and ICAM 2  [ **ICAM2** ]  but not E selectin, VLA 4, VCAM 1 or Mac 1.  Ref: 12454301 Thorax, 2002
